# HE4 as a biomarker for diagnosis of lung cancer: A meta-analysis

**DOI:** 10.1097/MD.0000000000017198

**Published:** 2019-09-27

**Authors:** Yong-Peng He, Li-Xian Li, Jia-Xi Tang, Lin Yi, Yi Zhao, Hai-Wei Zhang, Zhi-Juan Wu, Hai-Ke Lei, Hui-Qing Yu, Wei-Qi Nian, Lin Gan

**Affiliations:** a Department of Biochemistry and Molecular Biology, College of Basic Medical sciences, Southwest Medical University, Luzhou; b Chongqing Key Laboratory of Translational Research for Cancer Metastasis and Individualized Treatment, Chongqing University Cancer Hospital & Chongqing Cancer Institute & Chongqing Cancer Hospital, Chongqing, China.

**Keywords:** biomarker, diagnosis, HE4, lung cancer, meta-analysis

## Abstract

**Background:**

The aim of our study was to assess the value of serum human epididymis protein 4 (HE4) to diagnose lung cancer and provide reliable scientific conclusions to guide clinical practice.

**Methods:**

A systematic search of the PubMed, EMBASE, Cochrane Library, Chinese National Knowledge Infrastructure, Chinese Biomedical Literature, and WANFANG databases was conducted to identify all studies examining serum HE4 in the diagnosis of lung cancer published up to June, 2017. The Quality Assessment of Diagnostic Accuracy Studies tool was used to evaluate the methodological quality of each trial. The meta-analysis was performed using STATA software and Review Manager 5.3.

**Results:**

There were 21 studies involving 1883 cases and 1696 controls included in our meta-analysis. The pooled sensitivity and specificity of HE4 for diagnosing lung cancer were 0.73 (95% confidence interval [CI] 0.68–0.78) and 0.86 (95% CI 0.81–0.91), respectively. The positive likelihood ratio and negative likelihood ratio were 5.4 (95% CI 3.8–7.5) and 0.31 (95% CI 0.26–0.37), respectively. The diagnostic odds ratio was 17 (95% CI 12–26). The area under the curve of the summary receiver-operating characteristic curve was 0.86 (95% CI 0.83–0.89). Race, assay method, type of cancer, sample size, and publication date might be sources of heterogeneity in our meta-analysis. Subgroup analyses showed that the sensitivity in Caucasians was higher than that in Asians (0.81, 95% CI 0.71–0.91; and 0.71, 95% CI 0.66–0.77, respectively), but the specificity in Asians was better than that in Caucasians (0.87, 95% CI 0.81–0.92; and 0.85, 95% CI 0.73–0.97, respectively). The chemiluminescent microparticle immunoassay had the highest sensitivity, with 0.79 (95% CI 0.73–0.97), and the enzyme-linked immunosorbent assay had the highest specificity, with 0.87 (95% CI 0.79–0.94). HE4 had high diagnostic efficacy when screening for small cell lung cancer with the highest specificity (0.90, 95% CI 0.77–1.00).

**Conclusions:**

HE4 is a relatively promising and effective biomarker for the diagnosis of lung cancer. Furthermore, given the limitations of our study, additional large-scale and well-designed studies are needed in the future.

## Abstract 2

Supplemental Digital Content is available in the text

## 1 Introduction

Lung cancer is one of the most common cancers in China and worldwide, and it is also one of the leading causes of cancer mortality in both males and females.^[1–4]^ This disease is typically diagnosed at an advanced stage, and the 5-year net survival is 10% to 20% in most countries.^[4]^ Due to this high mortality, early detection may be a valuable approach to detect the disease at an earlier, asymptomatic, and potentially curable stage. Lung cancer may potentially be diagnosed at an early stage among high-risk individuals through the use of screening with low-dose computed tomography (LDCT), which can reduce lung cancer-related mortality. However, the positive outcome may generate new issues related to the rate of overdiagnosis of indolent cancer.^[5]^ Furthermore, people screened for lung cancer with LDCT comprise a small proportion.^[6]^ Currently, some serum tumor markers, such as carcinoembryonic antigen, squamous cell carcinoma-associated antigen, cytokeratin-19 fragment, neuron-specific enolase, and pro-gastrin-releasing peptide, can significantly improve the diagnosis of lung cancer, but specific markers are still lacking.^[7–9]^

Human epididymis protein 4 (HE4), encoded by the WAP 4-disulfilde core domain 2 (WFDC2) gene, is a promising biomarker for ovarian cancer.^[10]^ This molecule has been approved by the US Food and Drug Administration for use in the United States to monitor ovarian cancer for disease recurrence, differential diagnosis, and malignancy likelihood assessment in women with a pelvic mass.^[11,12]^ In recent years, an increasing number of clinical studies have shown that HE4 has a high diagnostic capacity for lung cancer.^[13–23]^ However, studies on HE4 are mostly from individual research centers, and the results of evidence-based medicine from different research centers are lacking. Therefore, we conducted a meta-analysis based on relevant and available studies to assess the value of serum HE4 for diagnosing lung cancer and provide reliable scientific conclusions to guide clinical practice.

## 2 Methods

### 2.1 Search strategy and study selection

A systematic search of the PubMed, EMBASE, Cochrane Library, Chinese National Knowledge Infrastructure, Chinese Biomedical Literature, and WANFANG databases was conducted to identify relevant studies published up to June, 2017. The search strategy used both medical subject heading terms and free-text words to increase the sensitivity of the search. The search words were as follows: (“HE4” or “human epididymis protein 4” or “whey-acidic-protein four-disulfide core protein-2” or “WFDC2”) and (“nsclc” or “non-small cell lung cancer” or “non-small cell lung carcinoma” or “lung carcinoma” or “lung squamous cell carcinoma” or “adenocarcinoma of lung” or “squamous cell carcinoma of lung” or “lung cancer” or “lung neoplasms” or “lung tumor”). Papers published in English and Chinese were included in our study. Authors of trial reports published only as abstracts and with incomplete data were contacted and asked to contribute full datasets or completed papers. Additionally, the bibliographies of all identified relevant studies were manually reviewed to potentially identify any additional studies that may have been missed by the electronic search. The strategy used for PubMed is shown in Supplementary Data 1.

Two investigators independently assessed the publication titles, abstracts, and full-text articles using predesigned eligibility forms according to the eligibility criteria. Any disagreement between investigators was resolved through consensus with a third investigator.

### 2.2 Study inclusion and exclusion criteria

In our meta-analysis, eligible studies had to meet the following standards: serum HE4 was used to detect patients with lung cancer as the case group and patients with benign lung diseases and/or healthy individuals as the control group; data such as the true positive (TP), false positive (FP), false negative (FN), and true negative (TN) were available in the studies; the measurement of serum HE4 must use commercial reagents; the literature reviewed was published in Chinese or English; if there were duplicated data, we chose the most complete data or the most recent data; the cut-off level must be presented. Excluded were the following standards: papers from which the extracted data were not sufficient; review articles, meta-analyses, meeting abstracts, case reports, and systematic reviews, and also preclinical studies; Studies with ambiguous diagnostic criteria.

### 2.3 Data extraction and quality assessment

All data were extracted independently from the studies by 2 investigators, including study characteristics (first author, publication year, country, assay method, type of cancer, cut-off point), and number of samples and outcome data (TP, FP, FN, and TN). The methodological quality of each trial was evaluated by the Quality Assessment of Diagnostic Accuracy Studies (QUADAS-2) tool and Review Manager5.3 (The Nordic Cochrane Center, The Cochrane Collaboration, 2014). According to the Cochrane guidelines, high, unclear, or low risk of bias of the patient selection, index tests, reference standards, and flow and timing domains were evaluated. Applicability concerns in the patient selection, index tests, and reference standards were also evaluated.

### 2.4 Statistical analysis

A bivariate regression model was used to calculate the pooled sensitivity, specificity, negative likelihood ratio (NLR), positive likelihood ratio (PLR), diagnostic odds ratio (DOR), area under the curve (AUC), and associated 95% confidence intervals (CIs). Spearman rank correlation analysis was used to test the threshold effect. Inconsistency index (*I*^2^), a chi-square test, and a bivariate box-plot were used to assess heterogeneity. Studies with an *I*^2^ statistic of 25% to 50% were considered to have low heterogeneity, those with an *I*^*2*^ statistic of 50% to 75% were considered to have moderate heterogeneity, and if *I*^2^ > 75%, high heterogeneity was considered to exist in the studies. A random-effects model was used for the meta-analysis if heterogeneity was present. Otherwise, a fixed-effects model was applied. In addition, to investigate the potential effect of heterogeneity, we carried out meta-regression and subgroup analyses. We used a likelihood ratio scatter-gram to evaluate the confirmation and exclusion capacities of HE4. A Fagan diagram was employed to calculate the post-test probability. Finally, Deek funnel plot was used to assess the publication bias. All statistical analyses were performed using STATA software (STATA version 12.0, Stata Corporation).

## 3 Results

### 3.1 Literature research and characteristics of the studies

**Figure 1:**
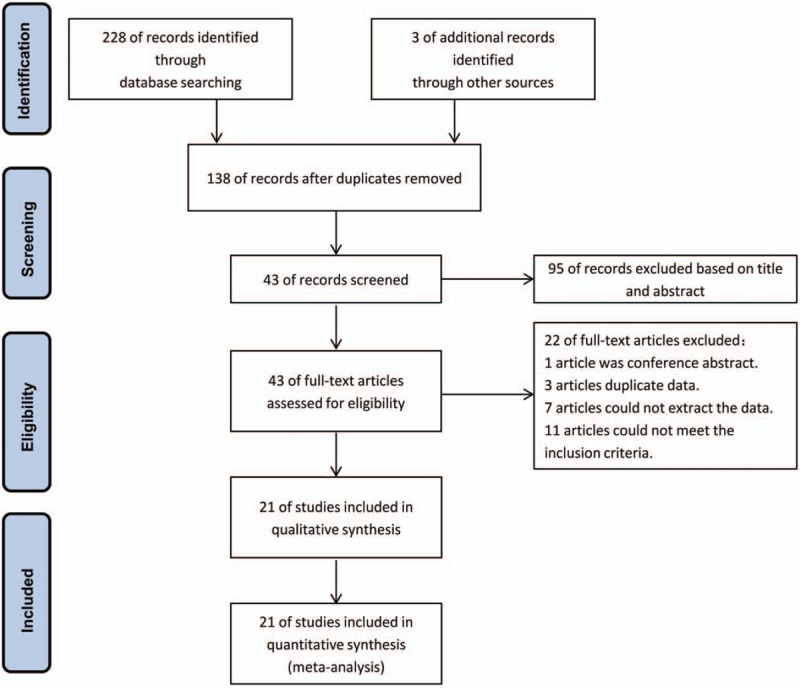
Flow chart of the study selection process.

A total of 228 literature citations were identified by the initial database search, and 3 citations were identified through other sources. A total of 93 records were excluded because of duplicate studies, and 95 records were excluded based on titles and abstracts. The remaining 43 full-text articles were reviewed for a more detailed evaluation, and 22 of them were also excluded because 1 article was a conference abstract, 3 articles were duplicate data, 7 articles did not provide data, and 11 articles did not meet the inclusion criteria. Finally, 21 studies that met the inclusion criteria were included in our meta-analysis. The flow chart of the study selection process is shown in Fig. 1.

The major characteristics of the included studies are shown in Table 1. There were 3579 samples from 6 different countries included in our meta-analysis involving 1883 cases and 1696 controls. The sample size ranged from 70 to 434. All studies were published between 2012 and 2017. A total of 10 studies were published in English, and 11 were published in Chinese. Four studies examined a Caucasian population, and 17 studies had an Asian population. The HE4 cut-off levels were reported in these studies with different units. Three different methods were used to detect the level of HE4: 9 of the 21 studies used enzyme-linked immunosorbent assay (ELISA); chemiluminescent microparticle immunoassay (CMIA) were used in 4 studies; and 8 studies used electro-chemiluminescence immunoassay (ECLIA).

**Table 1:**
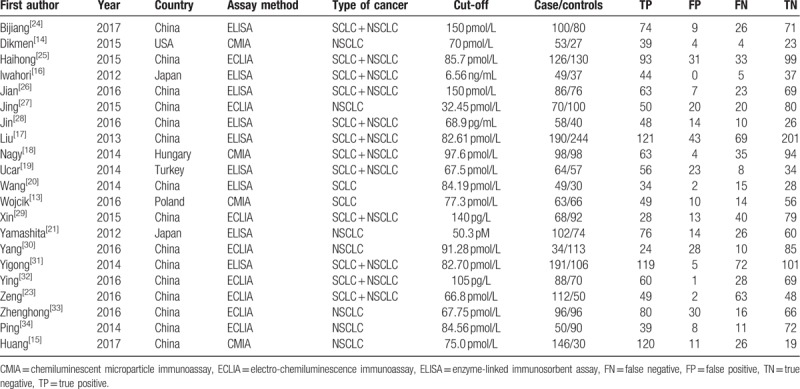
The major characteristics of the included studies.

### 3.2 Quality assessment

**Figure 2:**
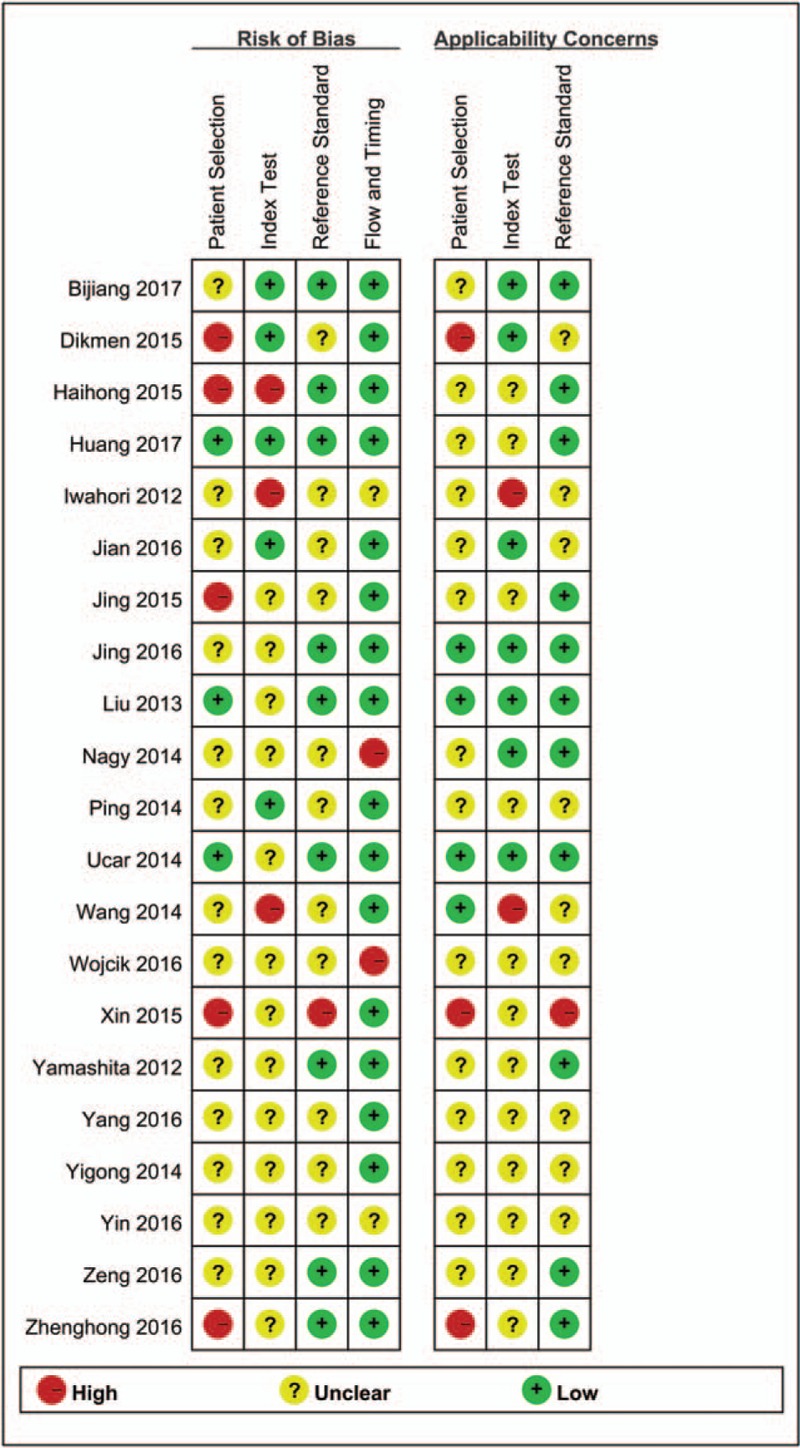
Methodological quality of the study on HE4 for the diagnosis of lung cancer. HE4 = human epididymis protein 4.

According to QUADAS-2, the methodological quality assessment of each trial is shown in Fig. 2. The risk of bias in patient selection was high in 5 studies. Three studies were shown to have a high bias in their index tests, and only 1 had a high bias in the reference standard. Seventeen studies were found to have a low bias in their flow and timing. Three studies showed a high bias in patient selection in the applicability concern, 2 studies were shown to have a high bias in the index test, and only 1 study showed a high bias in the reference standard. The assessment of the quality of most of the included studies was not bad, but some studies were evaluated as high risk in patient selection, index test, reference standard, flow and timing for risk, and bias or applicability concern, which might impact the pooled effects.

### 3.3 Meta-analysis

**Figure 3:**
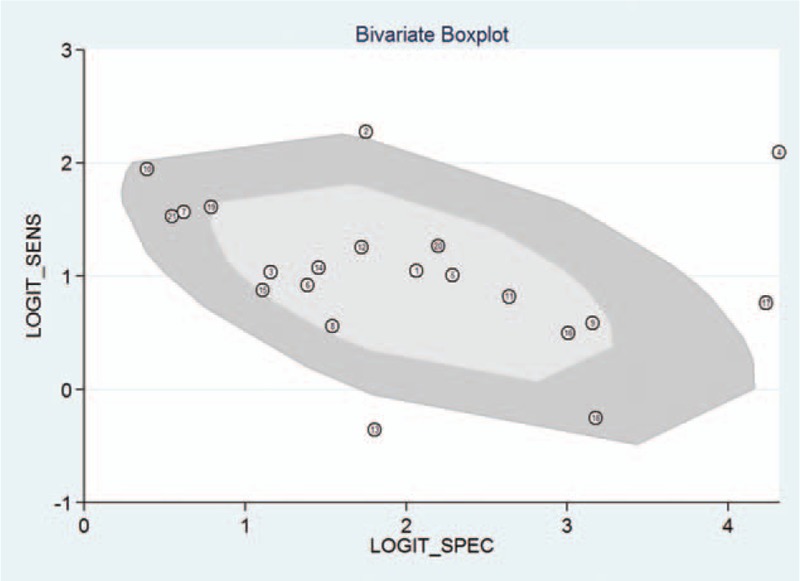
Bivariate box-plot assessing heterogeneity of 21 included trials.

**Figure 4:**
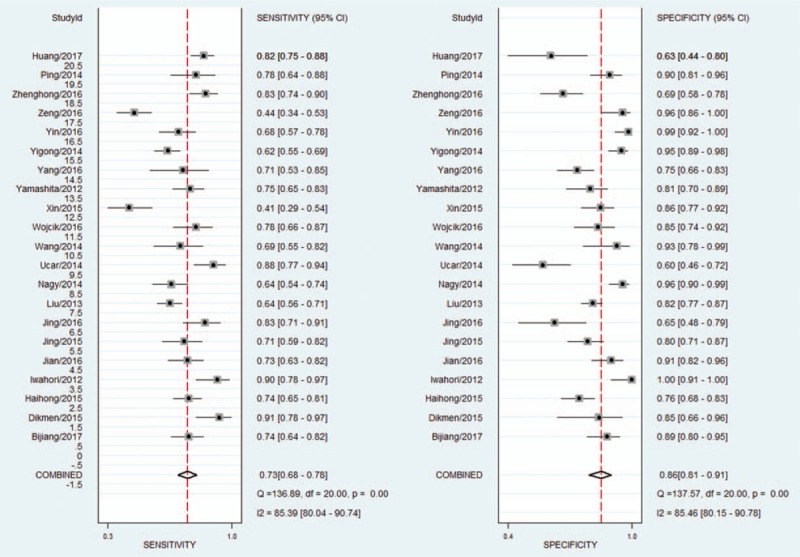
Forest plot of the sensitivity and specificity for HE4 in the diagnosis of lung cancer. HE4 = human epididymis protein 4.

**Figure 5:**
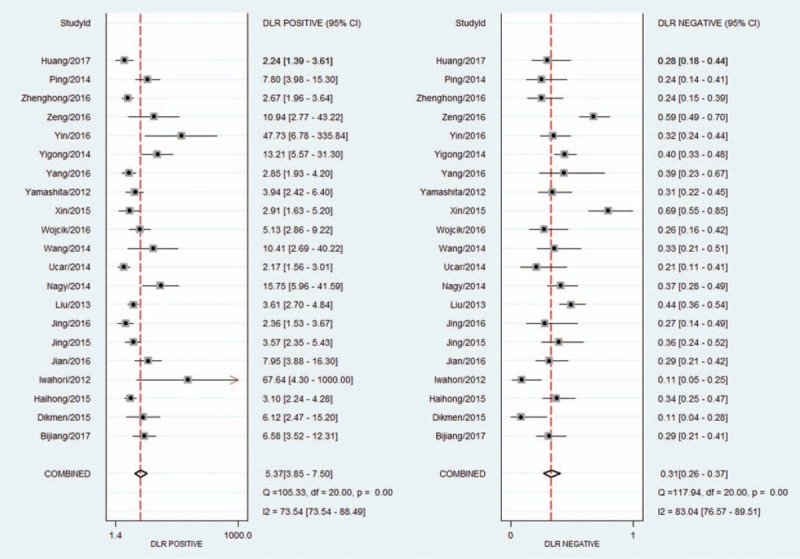
Forest plot of the PLR and NLR for HE4 in the diagnosis of lung cancer. HE4 = human epididymis protein 4, NLR = negative likelihood ratio, PLR = positive likelihood ratio.

**Figure 6:**
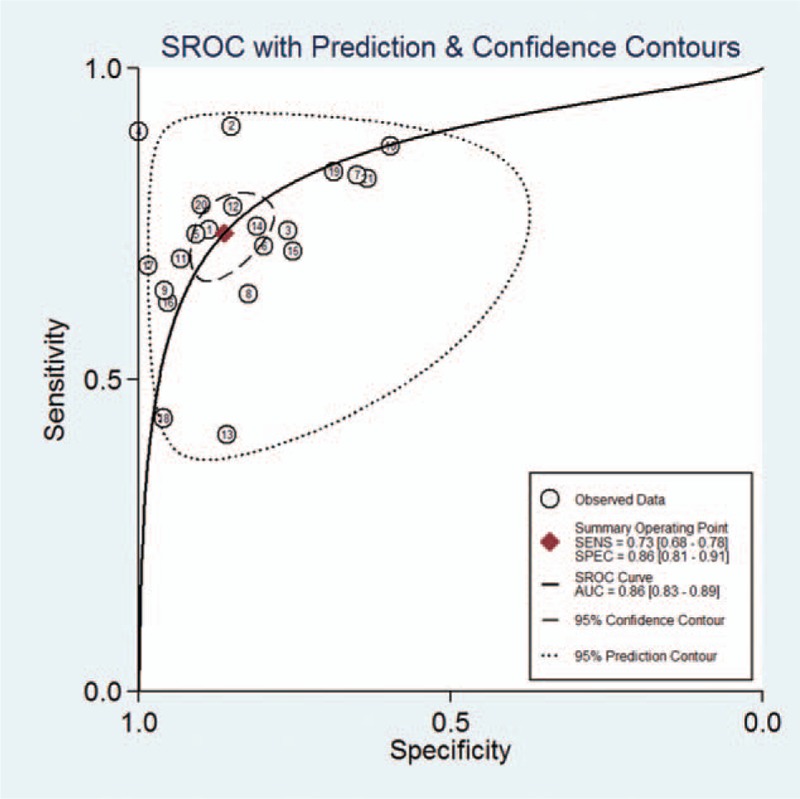
SROC curve for HE4 in the diagnosis of lung cancer. AUC = area under the cure, HE4 = human epididymis protein 4, SROC curve = summary receiver-operating characteristic curve.

**Figure 7:**
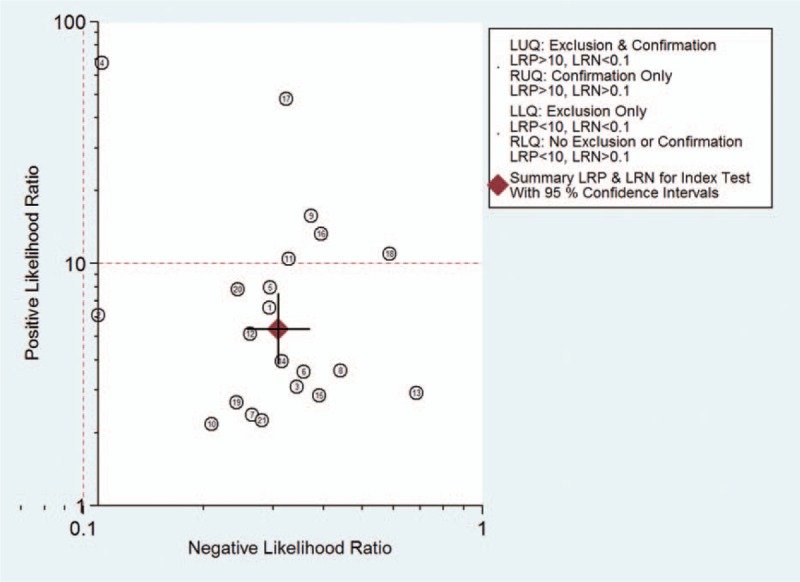
Likelihood ratio scatter-gram evaluating the confirmation and exclusion capacity of HE4 for diagnosing lung cancer. HE4 = human epididymis protein 4.

**Figure 8:**
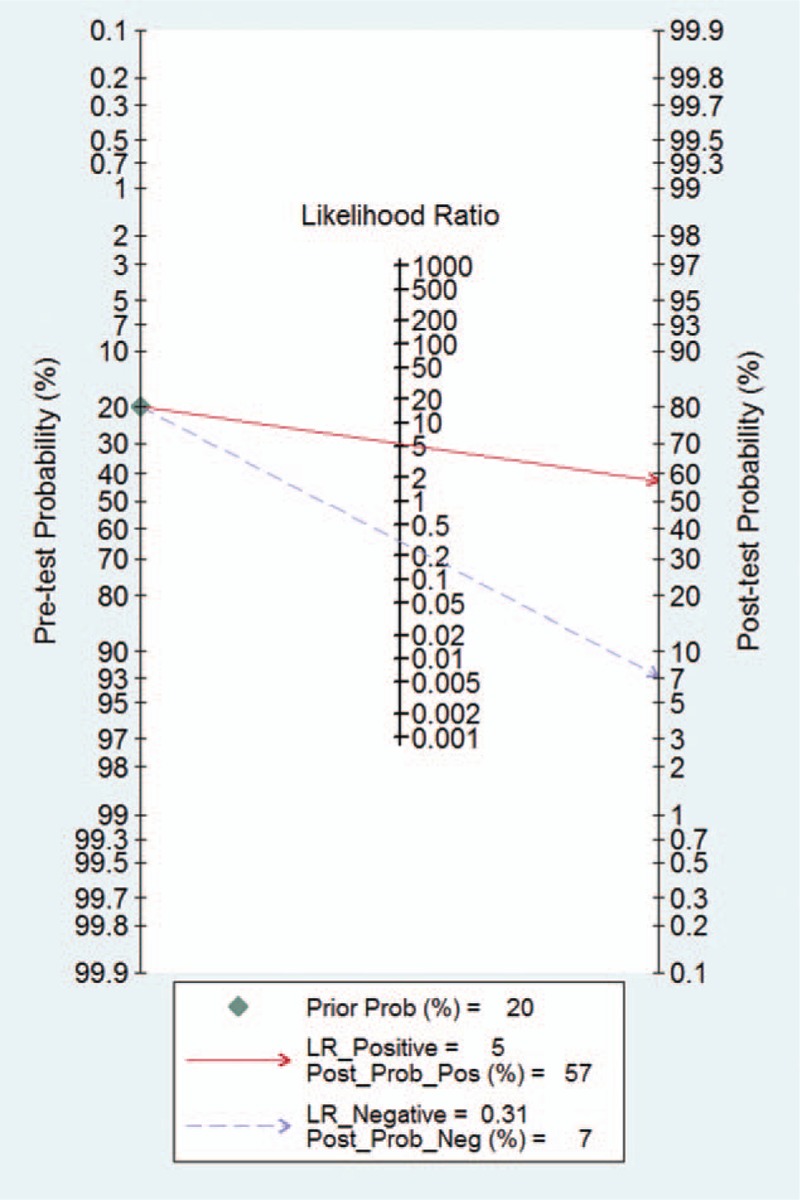
Fagan diagram of HE4 for diagnosing lung cancer. HE4 = human epididymis protein 4.

**Figure 9:**
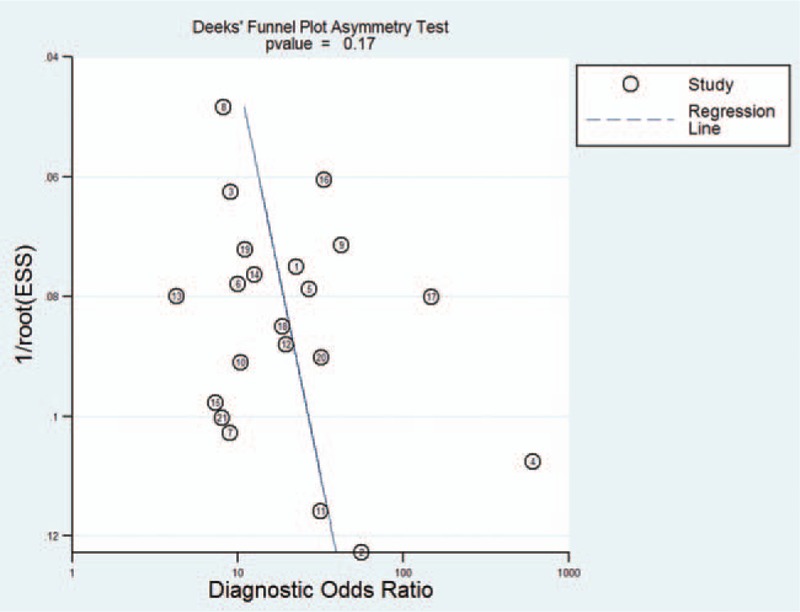
Deek funnel plot for the assessment of the publication bias.

The *I*^2^ of 98 (95% CI 97–99), chi-square test (*Q* = 119.859, *P* = .000), and a bivariate box-plot (Fig. 3) indicated that significant heterogeneity was present. Therefore, a random-effects model was performed for the meta-analysis in our study. The Spearman correlation coefficient was −0.54 (*P* = .29), suggesting that there was no significant threshold effect. The pooled sensitivity and specificity of HE4 for diagnosing lung cancer were 0.73 (95% CI 0.68–0.78) and 0.86 (95% CI 0.81–0.91), respectively (Fig. 4). The PLR and NLR were 5.4 (95% CI 3.8–7.5) and 0.31 (95% CI 0.26–0.37), respectively (Fig. 5). The DOR was 17 (95% CI 12–26). The AUC of the SROC was 0.86 (95% CI 0.83–0.89) (Fig. 6). According to the likelihood ratio scattergram, the confirmation and exclusion capacities of HE4 for diagnosing lung cancer were limited (Fig. 7). As shown by the Fagan diagram, the post-test probability corresponding to PLR and NLR was 57% and 7%, which differed substantially from the pretest probability (20%) (Fig. 8). To assess the publication bias for the diagnostic, we used Deek's funnel plot asymmetry test. There was no obvious asymmetry in the funnel plot, indicating no significant publication bias in this meta-analysis (*P* = 0.17) (Fig. 9).

### 3.4 Meta-regression and subgroup analyses

**Figure 10:**
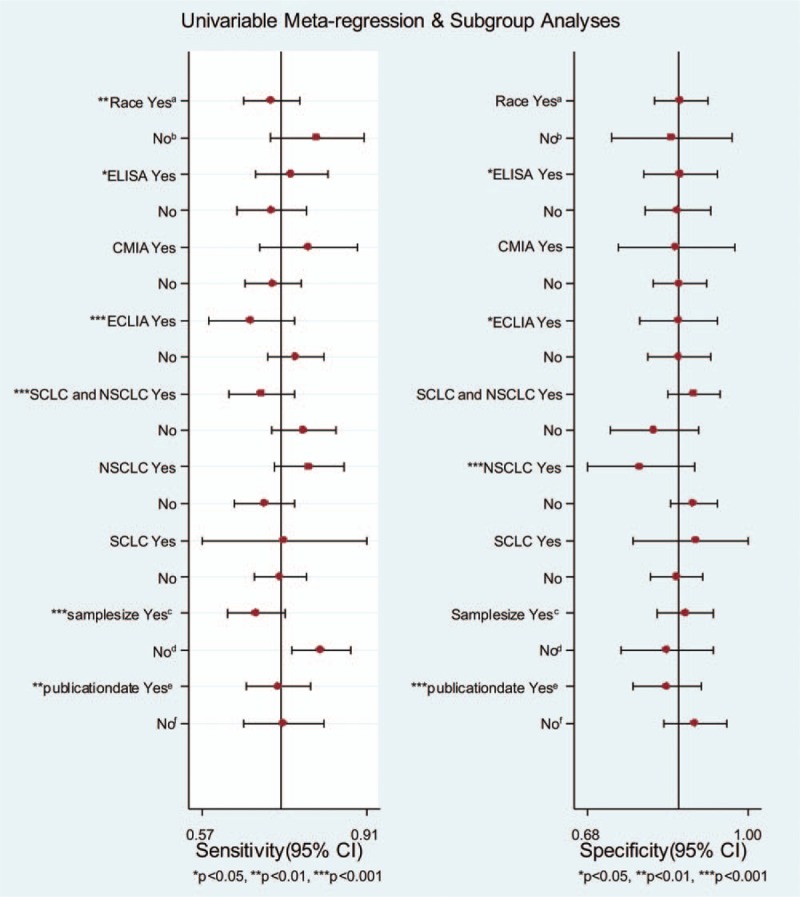
Forest plot of meta-regression and subgroup analyses of sensitivity and specificity for HE4 in diagnosing lung cancer. (A) Asian; (B) Caucasian; (C) total patients ≥150 cases; (D) total patients <150 cases; (E) publication year ≥2014; (F) publication year <2014. HE4 = human epididymis protein 4, NSCLC = nonsmall cell lung cancer, SCLC = small lung cancer.

Because heterogeneity existed in our study, univariable meta-regression and subgroup analyses were carried out to investigate potential sources of heterogeneity. Race, assay method (ELISA, CMIA ECLIA), type of cancer (small cell lung cancer [SCLC] and nonsmall cell lung cancer [NSCLC], SCLC, NSCLC), sample size, and publication date were included in the meta-regression analysis of sensitivity and specificity (Fig. 10). The forest plot of the univariable meta-regression indicated that race, assay method (ELISA, ECLIA), type of cancer (SCLC and NSCLC), sample size, and publication date may be the sources of the heterogeneity in the sensitivity, whereas assay method (ELISA, ECLIA), type of cancer (NSCLC), and publication date may be the sources of the heterogeneity in the specificity in our meta-analysis.

Race, assay method, and type of cancer were included in the subgroup analyses (Table 2). The sensitivity in Caucasians was higher than that in Asians (0.81, 95% CI 0.71–0.91; and 0.71, 95% CI 0.66–0.77, respectively), but the specificity in Asians was better than that in Caucasians (0.87, 95% CI 0.81–0.92; and 0.85, 95% CI 0.73–0.97, respectively). Regarding the assay method, when CMIA was used to detect HE4, the sensitivity was the highest at 0.79 (95% CI 0.73–0.97). When the ELISA was used, the specificity was the highest at 0.87 (95% CI 0.79–0.94). For the type of cancer, when HE4 was used to diagnose NSCLC, the sensitivity was highest at 0.79 (95% CI 0.72–0.87), and the specificity was highest in small cell lung cancer (SCLC) at 0.90 (95% CI 0.77–1.00).

**Table 2:**
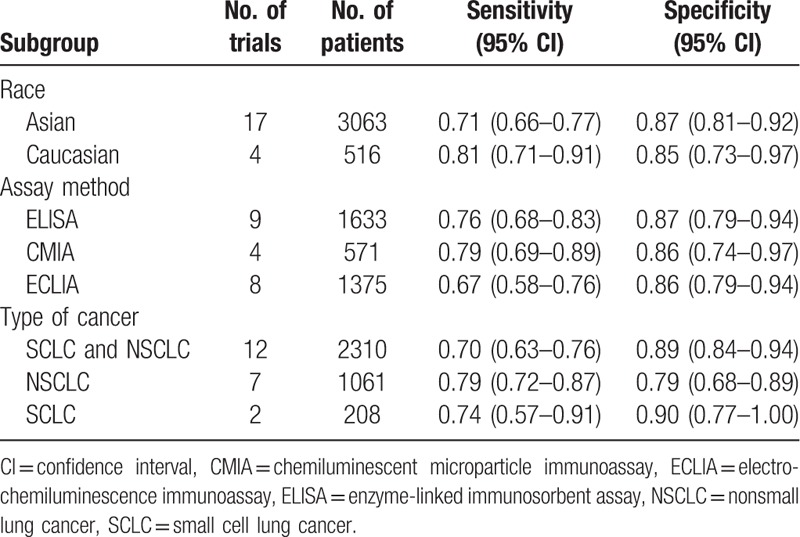
Subgroup analyses of race, assay method, and type of cancer.

## 4 Discussion

Human epididymis protein 4—a promising biomarker—has been commonly used in many malignant tumors, especially in ovarian cancer.^[11,35,36]^ The sensitivity and specificity of HE4 was higher than that of cancer antigen 125 as a tumor marker for ovarian cancer diagnosis. Accumulating evidence has demonstrated that HE4 could be used to diagnose lung cancer.^[14–21,23]^ However, to assess the value of HE4 for diagnosing lung cancer, the data of evidence-based medicine from different research centers should be subjected to pooled analysis. The impact of race, assay method, and type of lung cancer should be determined. The present meta-analysis showed that HE4 was highly useful for the differential diagnosis of lung cancer with good sensitivity and specificity, and it was a potential serum tumor marker.

A previous meta-analysis involving only 715 cases and 549 controls from 7 studies indicated that serum HE4 is a potential marker for lung cancer diagnosis.^[37]^ Using the previous meta-analysis as a base, we included 3579 samples from 21 studies in our meta-analysis involving 1883 cases and 1696 controls. The sample size increased nearly 3-fold compared with that of the previous meta-analysis. Moreover, 13 studies, including the present meta-analysis, were published after 2014. Therefore, the evidence on HE4 for diagnosing lung cancer in our study was strong. It was unfortunate that the source of heterogeneity was not detected because the number of eligible studies was limited in the previous meta-analysis. Our study addressed this issue to a degree.

To investigate potential sources of heterogeneity, we performed univariable meta-regression and subgroup analyses. Race, assay method, type of cancer, sample size, and publication date might be sources of heterogeneity in our meta-analysis. The sensitivity and specificity were investigated between Asian and Caucasian populations. The sensitivity of Caucasians for HE4 in diagnosing lung cancer was higher than that of Asians. Nevertheless, the specificity was better in Asians than Caucasians. Therefore, the diagnostic performance may be different in different races or regions. The diagnostic performance also differed depending on the different assay methods used to diagnose lung cancer for HE4. ELISA, CMIA, and ECLIA were investigated in our subgroup. CMIA had the highest sensitivity, whereas ELISA had the highest specificity. Therefore, it is difficult to find a method with the best sensitivity and specificity. It is worth noting that the sample size used for CMIA was small, with 571 samples. Future large studies should be performed to investigate the value of CMIA in detecting HE4 for diagnosis of lung cancer. To evaluate the value of HE4 in different types of lung cancer, we investigated the sensitivity and specificity of HE4 in different types of lung cancer. The sensitivity was the highest when using HE4 levels in the diagnosis of NSCLC, whereas HE4 had the highest specificity in SCLC. SCLC is a highly aggressive, lethal, and widely metastatic lung cancer accounting for approximately 15% of lung cancers. When diagnosed, this cancer is usually widely metastatic, and its 5-year overall survival rate is a mere 7%.^[38]^ However, the lack of high specificity markers to detect SCLC is even worse. In our study, the specificity was 0.90 (95% CI 0.77–1.00) for SCLC, demonstrating that HE4 would be a promising tool to screen for SCLC, although there were only 2 studies for SCLC included in our meta-analysis. The units of cut-off levels were diverse in our studies, so unified units should be recommended urgently, and the most suitable cut-off level should be confirmed.

Human epididymis protein 4 had a high sensitivity and specificity according to the present study. The Fagan diagram and the likelihood ratio scatter-gram revealed the clinical application value of HE4 for diagnosing lung cancer, although its application was limited in our study. The SROC has been recommended to assess the performance of a diagnostic test in a meta-analysis.^[39]^ Our meta-analysis found that the AUC of the SROC was 0.86 (95% CI 0.83–0.89), also demonstrating that HE4 was a potential biomarker for lung cancer diagnosis.

The present meta-analysis included 3579 samples from 21 studies obtained through a comprehensive search strategy. Meta-regression and subgroup analyses were performed to investigate sources of heterogeneity. However, our meta-analysis also had limitations. First, only papers published in English and Chinese were included in our meta-analysis, so articles in other languages may have been excluded, leading to unavoidable bias. Second, we did not evaluate the diagnostic value of HE4 for different stages of lung cancer for the lacking about this field. Further studies should focus on this issue. Third, there was no unified cut-off level, which was a limitation of the present meta-analysis. Finally, some studies included in our meta-analysis were evaluated as high risk in patient selection, index test, reference standard, flow and timing for risk, and bias or applicability concern, which might impact the results of our study.

## 5 Conclusions

The current study showed that HE4 was a relatively promising and effective biomarker for discriminating lung cancer patients from healthy individuals and benign lung disease patients, especially for SCLC. Furthermore, the diagnostic performance differed depending on the different assay method. CMIA had the highest sensitivity, and ELISA had the highest specificity. However, it is necessary to perform more large-scale and well-designed studies to confirm our conclusion.

## Author contributions

**Conceptualization:** Wei-qi Nian, Lin Gan.

**Data curation:** Yong-peng He, Lin Yi, Yi Zhao, Lin Gan.

**Formal analysis:** Yong-peng He, Hai-ke Lei.

**Funding acquisition:** Zhi-juan Wu, Hui-qing Yu.

**Investigation:** Hai-wei Zhang.

**Project administration:** Jia-xi Tang, Lin Gan.

**Resources:** Zhi-juan Wu.

**Software:** Li-xian Li.

**Supervision:** Lin Gan.

**Validation:** Wei-qi Nian.

**Writing – original draft:** Yong-peng He.

**Writing – review & editing:** Wei-qi Nian.

## Supplementary Material

Supplemental Digital Content
