# Supplementary material for: HE4 as a biomarker for diagnosis of lung cancer: A meta-analysis
Source: Medicine (Baltimore). 2019 Sep 27;98(39):e17198. doi: 10.1097/MD.0000000000017198 (PMC6775374; doi:10.1097/MD.0000000000017198)
Supplement: Supplemental Digital Content [file medi-98-e17198-s001.docx]

Supplementary Data 1: The strategy of PubMed

| Search | Query | Items found | Time |
| --- | --- | --- | --- |
| #20 | Search ((((((HE4) OR human epididymis protein 4) OR whey-acidic-protein four-disulfide core protein-2) OR WFDC2) OR "WFDC2 protein, human" [Supplementary Concept])) AND (((((((((((nsclc) OR non-small cell lung cancer) OR non-small cell lung carcinoma) OR lung adenocarcinoma) OR lung squamous cell carcinoma) OR adenocarcinoma of the lung) OR squamous cell carcinoma of the lung) OR lung cancer) OR Lung Neoplasms) OR lung tumor) OR "Lung Neoplasms"[Mesh]) | 40 | 10:01:32 |
| #19 | Search ((((((((((nsclc) OR non-small cell lung cancer) OR non-small cell lung carcinoma) OR lung adenocarcinoma) OR lung squamous cell carcinoma) OR adenocarcinoma of the lung) OR squamous cell carcinoma of the lung) OR lung cancer) OR Lung Neoplasms) OR lung tumor) OR "Lung Neoplasms"[Mesh] | 309979 | 10:00:55 |
| #18 | Search "Lung Neoplasms"[Mesh] | 199757 | 10:00:18 |
| #17 | Search lung tumor | 278396 | 9:59:27 |
| #16 | Search Lung Neoplasms | 243756 | 9:59:16 |
| #15 | Search lung cancer | 288859 | 9:58:34 |
| #14 | Search squamous cell carcinoma of the lung | 24819 | 9:58:20 |
| #13 | Search adenocarcinoma of the lung | 50719 | 9:58:10 |
| #12 | Search lung squamous cell carcinoma | 24819 | 9:57:59 |
| #11 | Search lung adenocarcinoma | 10666 | 9:57:45 |
| #10 | Search non-small cell lung carcinoma | 51516 | 9:57:34 |
| #9 | Search non-small cell lung cancer | 64021 | 9:57:23 |
| #8 | Search nsclc | 52106 | 9:57:14 |
| #7 | Search ((((HE4) OR human epididymis protein 4) OR whey-acidic-protein four-disulfide core protein-2) OR WFDC2) OR "WFDC2 protein, human" [Supplementary Concept] | 1090 | 9:56:52 |
| #6 | Search "WFDC2 protein, human" [Supplementary Concept] | 272 | 9:56:23 |
| #4 | Search WFDC2 | 298 | 9:55:53 |
| #3 | Search whey-acidic-protein four-disulfide core protein-2 | 1 | 9:55:44 |
| #2 | Search human epididymis protein 4 | 792 | 9:53:18 |
| #1 | Search HE4 | 497 | 9:53:11 |
